# Unveiling the role of ANGPT2 in esophageal cancer: A prognostic factor and potential oncogene

**DOI:** 10.3892/or.2026.9098

**Published:** 2026-03-12

**Authors:** Zhigang Zhai, Zhenyu Xue, Yuan Fang, Yixu Fan, Shijiao Li, Yunpeng Yu, Chuansheng Chen, Kaijun Zhang, Deyu Chen, Xiang Tang, Rui Ling

**Affiliations:** 1 Thoracic Oncology Department, The Affiliated Hospital of Jiangsu University, Zhenjiang, Jiangsu 212001, P.R. China; 2 Head and Neck Oncology Department, Affiliated Hospital of Jiangsu University, Zhenjiang, Jiangsu 212001, P.R. China; 3 Abdominal Oncology Department, Affiliated Hospital of Jiangsu University, Zhenjiang, Jiangsu 212001, P.R. China

**Keywords:** angiopoietin-2, esophageal cancer, prognosis, biomarker, immunity

## Abstract

Esophageal cancer (ESCA) is defined by high incidence and mortality rates. Emerging evidence implicates angiopoietin-2 (ANGPT2) as a key mediator in human carcinogenesis; however, its precise functional role and underlying mechanisms in ESCA remain incompletely elucidated. Clinical samples and The Cancer Genome Atlas, genotype-tissue expression and Gene Expression Omnibus databases were first used to analyze ANGPT2 expression, clinical relevance and prognostic value, demonstrating it as an independent prognostic factor and potential oncogene in ESCA. In addition, abnormal ANGPT2 expression affected ESCA migration and invasion *in vitro*. Subsequently, through correlation, expression and prognostic analyses, the upstream long-chain non-coding RNAs and microRNAs that regulate ANGPT2 were identified. In conclusion, gene set enrichment analysis revealed a connection between ANGPT2 and the immune system, with ANGPT2 level showing a strong positive correlation with tumor microenvironment cell infiltration, immune cell markers, and immune checkpoint expression. Furthermore, ANGPT2 expression was significantly associated with sensitivity to chemotherapeutic drugs. To summarize, overexpression of ANGPT2 in ESCA is associated with an unfavorable prognosis and compromised tumor immunity.

## Introduction

Esophageal cancer (ESCA) is the 6th leading cause of cancer-related mortality globally. Tens of thousands of individuals are diagnosed with ESCA each year (1). Despite significant progress in surgical, chemotherapy and radiation therapies for ESCA, the fatality rate remains elevated, with a five-year survival rate not exceeding 20% (2). An accumulating body of studies shows that numerous factors, such as smoking (3), alcohol consumption (4), gastroesophageal reflux disease (5), the immune system (6) and obesity (7), are related to the initiation and progression of ESCA. Accordingly, identifying promising biomarkers for ESCA diagnosis and prognosis is urgent.

Angiopoietins (ANGPT) represent a family of secreted factors that include ANGPT1/2/3/4 (in humans) (8–10). ANGPT2 is an antagonistic ligand that maintains the constitutively static endothelial ANGPT1/TIE2 signaling axis and has been extensively studied as a candidate molecule for second-generation anti-angiogenesis drugs (11). ANGPT2 is not only strongly associated with cardiovascular disease (CVD) but has also been established as a contributor to the initiation and progression of lung (12), colon (13), melanoma (14), gastric (15) and renal cell carcinoma (16). Beyond its angiogenic functions, ANGPT2 plays a documented role in immune modulation within the tumor microenvironment (TME). Elevated serum ANGPT2 levels correlate with therapeutic resistance, as they promote both proangiogenic and immunosuppressive activity (17). Additionally, ANGPT2 sensitizes TIE2^+^ M-myeloid-derived suppressor cells (MDSCs) to impede TAA-specific T cells (18). In addition, ANGPT2 and VEGFA have been reported to modulate ligand programmed cell death ligand 1 (PD-L1) (19). However, studies on the prognostic value and mechanism of ANGPT2 in ESCA are still lacking. Additionally, the relationship between ANGPT2 and tumor immunity in ESCA remains unclear.

In the present study, ANGPT2 expression and its association with survival prediction were first analyzed in various tumors. Subsequently, the expression of ANGPT2 was assessed in various ESCA databases and ESCA clinical samples, examining its clinical relevance and prognosis. An *in vitro* experiment was then conducted to illustrate the role of ANGPT2 in ESCA, further exploring the upstream regulatory factors of ANGPT2 to determine possible regulatory microRNAs (miRNAs or miRs) and long non-coding RNAs (lncRNAs). Finally, gene set enrichment analysis (GSEA) suggested that ANGPT2 is related to immunity and subsequently revealed the interconnection between ANGPT2 expression and TME infiltrating characteristics, immune cell (IC) markers, and immune checkpoints in ESCA. Furthermore, the interaction between ANGPT2 and chemotherapeutic drug sensitivity was analyzed. The findings of the present study indicate that in patients with ESCA, high expression of ANGPT2 is involved in tumor immunity and chemotherapeutic drug sensitivity and suggest a poor prognosis for patients.

## Materials and methods

### Patients and tumor tissues

In the present study, a retrospective analysis was performed on 48 tumors and 21 surrounding tissues [collected between January 2014 and December 2016 at the Affiliated Hospital of Jiangsu University (Zhenjiang, China)] acquired from patients with ESCA who did not receive chemotherapy or radiotherapy. The present study was approved (approval no. KY201901) by the Ethics Committee of The Affiliated Hospital of Jiangsu University (Zhenjiang, China). Before undergoing surgical resection, all patients signed an informed consent form. Furthermore, apart from the verification by pathologists working independently, tissue samples were preserved in liquid nitrogen at −80°C until they were utilized for reverse transcription-quantitative PCR (RT-qPCR) and western blotting. The inclusion and exclusion criteria for the selection of patient samples with ESCA was listed as follows:

Inclusion criteria: (i) Aged ≥18 years old, with no sex restriction; (ii) diagnosed with primary ESCA by histopathological examination at the Affiliated Hospital of Jiangsu University from 2014 to 2016, with a clear and confirmed pathological diagnosis; (iii) scheduled for surgical resection of ESCA, without receiving neoadjuvant chemotherapy, radiotherapy, targeted therapy, immunotherapy or other antitumor treatments before surgery; (iv) no history of malignant tumors, and no primary malignant tumors in other organs or systems; (v) voluntarily signed a written informed consent form before surgical treatment and agreed to participate in the present study; (vi) tumor tissue samples and paired adjacent normal esophageal tissue samples were successfully collected, and the sample quality was verified to be qualified by independent pathologists, which could be used for subsequent experimental detection such as RT-qPCR, western blotting and immunohistochemistry (IHC).

Exclusion criteria: (i) Under 18 years old; (ii) unclear pathological; (iii) diagnosis, or diagnosed as metastatic tumors involving the esophagus rather than primary ESCA; (iv) complicated with primary malignant tumors in other organs or systems, or with a history of malignant tumors; (v) the collected tumor tissue or adjacent normal tissue samples were contaminated, degraded, or insufficient in quantity, which could not meet the requirements of subsequent experimental detection; (vi) refused to sign the informed consent form, or voluntarily withdrew from the study during the research process; (vii) complicated with severe underlying diseases such as severe liver and kidney failure, serious cardiovascular and cerebrovascular diseases, uncontrolled infectious diseases, which may affect the collection of clinical samples and the judgment of research results.

### Cell lines

Cell lines TE-1 (cat. no. ZQ0235), KYSE30 (cat. no. ZQ09963) and KYSE150 (cat. no. ZQ0449; all from Shanghai Zhongqiao Xinnzhou Biotechnology Co., Ltd.) and the immortalized normal esophageal squamous epithelium (Het-1A; cat. no. BNCC342346; BeNa Culture Collection) were authenticated using Short Tandem Repeat analysis. All cell lines were used for experiments at passage numbers 8–15 to ensure consistent phenotypic and functional stability. Mycoplasma contamination was routinely screened using the Myco-Visible Mycoplasma LAMP Detection Kit (MP Biomedicals, LLC) targeting the conserved 16S rRNA gene, and all cell lines tested negative before experimentation. TE-1, KYSE30, KYSE150, 293T and Het-1A cell lines were cultured in RPMI-1640 and DMEM media with 10% fetal bovine serum (FBS; Biological Industries) and 100 IU/ml of penicillin and streptomycin (all from Gibco; Thermo Fisher Scientific, Inc.) and incubated at 37°C in 5% CO_2_.

### Analysis of RT-qPCR using quantitative methods

Total RNA isolation from ESCA tissues and cells was performed using the TRIzol reagent (Takara Biotechnology Co., Ltd.) according to the manufacturer's protocols. To measure relative RNA levels, the BioMate 3S Analyzer (Thermo Fisher Scientific, Inc.) was used. The next step involved performing RT-qPCR reactions using a reverse transcription kit, along with Cham Q SYBR master mix (both from Vazyme Biotech Co., Ltd.). The listed primers were as follows: ANGPT2 forward, 5′-AACTTTCGGAAGAGCATGGAC-3′ and reverse, 5′-CGAGTCATCGTATTCGAGCGG-3′; and β-actin forward, 5′-TCACCCACACTGTGCCCATCTACGA-3′ and reverse 5′-CAGCGGAACCGCTCATTGCCAATGG-3′. The thermal cycling conditions for RT-qPCR were set as follows: initial denaturation at 95°C for 30 sec; followed by 40 cycles of denaturation at 95°C for 10 sec and annealing/extension at 60°C for 30 sec; a subsequent melting curve analysis was conducted (95°C for 15 sec, 60°C for 1 min, and 95°C for 15 sec) to verify the specificity of the PCR amplicons, with all procedures performed on a real-time PCR detection system. Then, the 2^−ΔΔCq^ methodology was applied to calculate the relative expression of ANGPT2, using β-actin as an internal reference gene (20). Each experiment had three identical holes, and all experiments were conducted thrice.

### Western blotting

Cell lysis was performed in RIPA buffer containing PMSF (Beijing Solarbio Science Technology Co., Ltd.) according to the protocol. A BCA protein assay (CoWin Biosciences) was employed to determine protein concentration, with the absorbance measured at 562 nm using a Multiskan SkyHigh Microplate Spectrophotometer (Thermo Fisher Scientific, Inc.,) and protein concentration calculated via SkanIt Software 6.0 (Thermo Fisher Scientific, Inc.). Equal amounts of protein (20 µg) were loaded per lane and separated using 8 and 12% SDS-PAGE and then transferred to a PVDF membrane. A 1-h blockage of the membrane was conducted at room temperature using a 5% BSA solution (Beijing Solarbio Science Technology Co., Ltd.) in TBST containing 0.1% Tween-20 (Beijing Solarbio Science Technology Co., Ltd.). The membranes were then incubated overnight at 4°C with primary antibodies diluted in TBST containing 5% BSA: ANGPT2 (1:1,000; cat. no. sc-74403; Santa Cruz Biotechnology, Inc.) and GAPDH (1:1,000; cat. no. sc-47724; Santa Cruz Biotechnology, Inc.). After washing with TBST three times (10 min each), the membranes were placed for 1 h at room temperature with HRP-conjugated anti-rabbit or anti-mouse antibody (1:5,000; cat. nos. 7074P2 and 7076P2, respectively; Cell Signaling Technology, Inc.), subjected to enhanced ECL reagent (Vazyme Biotech Co., Ltd.), and images were captured using a Chemis Scope-4300 imager (Clinx Science Instruments Co., Ltd.).

### Histological analysis

IHC staining was conducted on formalin-fixed, paraffin-embedded tissue sections (4-µm-thick) according to established protocols. Briefly, the incubation of sections was conducted with a primary mouse monoclonal anti-ANGPT2 (1:50; Beyotime Institute of Biotechnology; 0.5% BSA/PBS) and with an HRP-conjugated anti-mouse secondary antibody (1:200; cat. no. NB7539; R&D Systems, Inc.; 0.5% BSA/PBS). Antigen-antibody complexes were observed with the DAB Substrate Kit (Abcam). Stained slides were initially screened at low magnification (×100) to determine the regions with the highest vascular density. A total of four representative fields per slide were selected and independently evaluated in a blinded manner by two investigators using light microscopes (Olympus Corporation) coupled with Image-Pro Plus 6.0 software (Media Cybernetics, Inc.). Staining intensity was scored as: 0 (negative), 1 (weak), 2 (moderate), or 3 (strong). The positively stained cell proportion was scored as follows: 0 (0%), 1 (<50%), 2 (50–75%), or 3 (>75%). The final IHC score was computed by multiplying the intensity and proportion scores. Tumors were classified as exhibiting low ANGPT2 expression if the score was <6, and high expression if ≥6.

### Small interfering RNA (siRNA) transfection

The siRNAs for ANGPT2 and a negative control (NC) at a final concentration of 50 nM which were then introduced into TE-1 cells and KYSE150 cells via Lipofectamine 2000 (Thermo Fisher Scientific, Inc.) at 37°C for 6 h, were provided by Shanghai Gene Pharma Co., Ltd. After transfection, the cells were cultured in complete medium for an additional 48 h before subsequent experimentation, and the transfected cells were further treated with additional treatments for the required duration and then subjected to functional determination. The sequences of si-ANGPT2 and si-NC are listed as follows: si-ANGPT2-1 sense, 5′-GCAUUCUGCUGUAUCUCUACCAUUU-3′ and antisense 5′-AAAUGGUAGAGAUACAGCAGAAUGC-3′; si-ANGPT2-2: sense 5′-GGAGAAUAUUGGCUGGGAATT−3′ and antisense, 5′-UUCCCAGCCAAUAUUCUCCTT−3′; si-ANGPT2-3 sense, 5′-GCAUCUACACGUUAACAUUTT−3′ and antisense 5′-AAUGUUAACGUGUAGAUGCTT−3′; and si-NC sense, 5′-UUCUCCGAACGUGUCACGUUU-3′ and antisense, 5′-ACGUGACACGUUCGGAGAAUU-3′. All siRNAs were chemically modified with 2′-O-methyl (2′-OMe) to enhance stability and reduce off-target effects, with HPLC purity ≥98%.

### Cell migration and invasion assays

Following siRNA transfection, TE-1 cells and KYSE150 cells were harvested, resuspended, and plated at a density of 7×10^4^ cells/well into the top chambers (8-µm pore polycarbonate membrane; Corning, Inc.) in serum-free medium. The bottom chamber medium included 10% FBS as a chemoattractant. For invasion assays, membranes were pre-coated with 60 µg of Matrigel (Becton, Dickinson and Company) at 4°C and subsequently incubated at 37°C for 2 h to allow for gel solidification. Post-incubation at 37°C in a 5% CO_2_ incubator for 24 or 48 h, the cells were fixed with 4% paraformaldehyde at room temperature for 15 min and subsequently stained with 0.5% crystal violet at room temperature for 20 min. Migratory and invasive cells were quantified by counting five randomly selected microscopic fields/well under an inverted light microscope.

### Wound healing assay

The siRNA-transfected cells were plated in 6-well dishes until they reached 80–85% confluence. Afterward, a straight scratch was made with a 200-µl pipette tip, and then the cells were cultured in serum-free medium for 24 h. Images of cells were subsequently captured using an inverted light microscope (Olympus Corporation).

### Dual-luciferase reporter assay

For the dual-luciferase reporter assay, wild-type (WT) and mutant (MUT) sequences of ANGPT2 (NM_001118887.2), MAPKAPK5-AS1 (NR_015404.2), and SNHG1 (ENST00000535076.6) were first synthesized via PCR-based Accurate Synthesis (PAS) with full-length overlap primers. The MUT constructs harbored a targeted mutation of ‘AACTGGA’ to ‘TTGACCT’. These sequences were cloned into the psicheck2.0 vector (cat. no. ZVE1012; Zaiji Biotechnology; http://www.zolgene.com) at the *Xho I* and *NotI* restriction sites, inserted downstream of the *Renilla* luciferase (RLU) gene with Firefly luciferase (FLU) serving as an internal control. Recombinant vectors were transformed into DH5α competent cells, and positive clones were validated by *Xho-ApaI* double digestion and Sanger sequencing. 293T cells (cat. no. ZCL1005; Zaiji Biotechnology) were cultured in DMEM-H medium (cat. no. C11995500bt; Gibco; Thermo Fisher Scientific, Inc.) supplemented with 10% FBS (cat. no. P30-3302; PAN Biotech UK, Ltd.), 1X Penicillin-Streptomycin (cat. no. 15140122; Gibco; Thermo Fisher Scientific, Inc.), 1X L-Glutamine (cat. no. 25030-081; Gibco; Thermo Fisher Scientific, Inc.), 1X Sodium Pyruvate (cat. no. 11360-070; Gibco; Thermo Fisher Scientific, Inc.) and 1X MEM NEAA (cat. no. 11140-050; Gibco; Thermo Fisher Scientific, Inc.) at 37°C in a humidified atmosphere with 5% CO_2_. Cells were passaged at 80% confluence at a 1:3 ratio following trypsinization (0.25% Trypsin-EDTA; cat. no. 25200056; Gibco; Thermo Fisher Scientific, Inc.) and seeded into 6-well plates 24 h prior to transfection to achieve 60–70% confluency at the time of transfection. Transfection was performed using Lipofectamine3000 (cat. no. L3000-015; Invitrogen; Thermo Fisher Scientific, Inc.) with two concentration gradients of hsa-miR-145-5p mimic or miR-negative control (miR-NC; 10 µl/well, 20 µM). Experimental groups included WT/MUT recombinant vector + hsa-miR-145-5p, while control groups consisted of recombinant vector + miR-NC and empty psicheck2.0 vector + hsa-miR-145-5p/miR-NC. Culture medium was replaced with fresh complete medium 6 h post-transfection. A total of 48 h after transfection, cells were lysed with 1X Lysis Buffer (from dual-luciferase reporter assay kit; cat. no. DD1205, Vazyme Biotech Co., Ltd.), and luciferase activities were measured using a SpectraMax iD5 multi-mode microplate reader (Molecular Devices, LLC) with freshly prepared FLU working solution (150 µg/ml) and RLU working solution (100 µM). RLU activity was calculated as the ratio of RLU activity to FLU activity. All experiments were performed in triplicate, and statistical significance was determined by Student's t-test with P<0.05 considered to indicate a statistically significant difference.

### The Cancer Genome Atlas (TCGA) database download

Data on gene expression and associated prognostic and clinicopathological information were obtained by accessing TCGA (https://genome-cancer.ucsc.edu/) for the following cancer types: bladder urothelial carcinoma (BLCA), breast invasive carcinoma (BRCA), cholangiocarcinoma (CHOL), esophageal carcinoma (ESCA), glioblastoma multiforme (GBM), head and neck squamous cell carcinoma (HNSC), kidney renal clear cell carcinoma (KIRC), kidney renal papillary cell carcinoma (KIRP), liver hepatocellular carcinoma (LIHC), lung adenocarcinoma (LUAD), lung squamous cell carcinoma (LUSC), pancreatic adenocarcinoma (PRAD), rectum adenocarcinoma (READ), stomach adenocarcinoma (STAD), kidney chromophobe (KICH), thyroid carcinoma (THCA), colon adenocarcinoma (COAD) and uterine corpus endometrial carcinoma (UCEC). The fragments per kilobase million (FPKM) measurement was transformed to one million transcripts per kilobase (TPM), which represented the same transcript quantity as the microarray.

### Gene expression profiling interactive analysis (GEPIA) database analysis

Using TCGA and GTEx data, GEPIA (http://gepia.cancer-pku.cn/) was employed to analyze tumor and normal gene expression profiling and to analyze the survival of ANGPT2 in 7 human tumors, including overall survival (OS) and disease-free survival (DFS), with P<0.05 considered to indicate a statistically significant difference.

### Gene Expression Omnibus (GEO; http://www.ncbi.nlm.nih.gov/) database analysis

GEO databases, including GSE20347, GSE38129, GSE45670 and GSE70409, were deployed to analyze ANGPT2 mRNA levels between ESCA cancer and normal tissues. Single-cell sequencing data (GSE160269) were used to analyze ANGPT2 expression in the TME, with P<0.05 considered to indicate a statistically significant difference.

### Nomogram scoring system

A prognostic nomogram was constructed using clinical characteristics and risk scores derived from multivariate survival analysis, implemented using the ‘rms’ package. The nomogram's calibration was assessed by plotting the predicted probabilities of 1-, 3-, and 5-year OS against the corresponding observed survival rates, allowing for a visual evaluation of predictive accuracy across different time points.

### StarBase database download

StarBase [ENCORI: The Encyclopedia of RNA Interactomes (sysu.edu.cn)] is used to predict miRNAs upstream of ANGPT and IncRNAs upstream of miR-145-5p. The regulatory network of the predicted miRNAs and ANGPT2 was visualized using Cytoscape software (v3.9.1; Cytoscape Consortium; http://cytoscape.org/).

### GSEA

The study initially sorted all genes based on their correlation with ANGPT2 expression and employed GSEA to elucidate the notable disparity in survival rates between the high and low ANGPT2 groups. Each analysis involved executing 1,000 permutations on the genome. ANGPT2 expression was utilized as a phenotypic marker. Each phenotype was classified based on the enrichment pathways using the nominal p-value and the normalized enrichment score (NES).

### Evaluation of the immune infiltration and TME in ANGPT2 expression cohorts

Using single-sample GSEA (ssGSEA), 23 subgroups of infiltrating ICs were quantified in both expression groups to assess the tumor-infiltrating IC proportion in the TME. The ‘ESTIMATE’ was used to assess the TME scores (immune, stromal and estimated) of both groups.

### Assessment of the immune infiltration and TME between ANGPT2 expression groups

To evaluate the tumor-infiltrating immune cell (TIIC) proportion in the TME, ssGSEA was utilized to determine the abundance of 23 infiltrating IC subgroups from both expression groups. The ‘ESTIMATE’ was performed to assess the TME scores of both groups.

### Drug sensitivity analysis

To evaluate the differential sensitivity to chemotherapeutic agents between high-and low-ANGPT2 expression groups in ESCA, the pRRophetic algorithm was utilized to predict the half-maximal inhibitory concentration (IC_50_) values of clinically commonly used chemotherapeutic drugs for ESCA. Briefly, transcriptomic data (FPKM format) of patients with ESCA from TCGA database were first converted to TPM values for standardization, followed by calibration of the pRRophetic model using the Cancer Cell Line Encyclopedia (CCLE) dataset to ensure the reliability of IC_50_ predictions. The preprocessed TCGA-ESCA transcriptomic data were then input into the calibrated model to predict IC_50_ values of first-line chemotherapeutic drugs for ESCA, including cisplatin, docetaxel, doxorubicin, erlotinib, paclitaxel and vinorelbine. Statistical comparison of the predicted IC_50_ values between the two ANGPT2 expression groups was performed using the Mann-Whitney U test with Holm-Bonferroni correction for multiple comparisons, where a lower IC_50_ value indicates higher sensitivity to the corresponding chemotherapeutic drug.

### TIMER2.0 database analysis

The correlation between ANGPT2 expression in ESCA and IC infiltration level or immune checkpoint expression was analyzed using TIMER 2.0 [TIMER2.0 (comp-genomics.org)], with P<0.05 deemed to indicate a statistically significant difference.

### Statistical analysis

Statistical analyses were performed using R software (v.4.0.5; Foundation for Statistical Computing; http://www.r-project.org/) and GraphPad Prism (v.9.0.0; Dotmatics). The Wilcoxon rank-sum test (unpaired) or Wilcoxon signed-rank test (paired) was used to compare mRNA, miRNA, lncRNA, or protein expression levels between tumor and normal tissues. The relationship between ANGPT2 expression and clinicopathological characteristics was analyzed via logistic regression. Kaplan-Meier curves were plotted to visualize OS and DFS, with differences compared using the log-rank test. The Cox proportional hazards model was used for univariate and multivariate analyses to identify independent prognostic factors, with the proportional hazards' assumption verified using Schoenfeld residuals. Spearman's correlation coefficient was applied for correlation analyses between ANGPT2 expression and immune cell infiltration, immune checkpoints, or target miRNAs/lncRNAs.

For multiple comparison analyses, appropriate correction methods were applied to control the family-wise error rate (FWER) or false discovery rate (FDR): (i) When comparing ANGPT2 expression across multiple tumor types (TCGA pan-cancer analysis) or among different clinicopathological subgroups (for example, tumor grade and lymph node status), Bonferroni correction was used to adjust the raw P-values for FWER. For correlation analyses involving multiple miRNAs/lncRNAs or multiple immune cell markers/immune checkpoints, Benjamini-Hochberg (BH) correction was implemented to control the FDR. (ii) In drug sensitivity analysis, Holm-Bonferroni correction was adopted for comparing IC_50_ values of multiple chemotherapeutic agents.

For *in vitro* functional assays and western blotting, data were presented as the mean ± SD from at least three independent biological replicates. Outliers were excluded using the Grubbs test (α=0.05), and differences between groups were assessed by unpaired Student's t-test or Mann-Whitney U test, with appropriate multiple comparison correction as specified. ssGSEA for immune infiltration was performed with 1,000 permutations, and nomogram calibration was evaluated using the Hosmer-Lemeshow test and C-index (bootstrap resampling, n=1,000). P<0.05 was considered to indicate a statistically significant difference, with all P-values adjusted for multiple comparisons unless otherwise stated.

## Results

### Pan-cancer analysis of ANGPT2 expression

**Figure 1.**
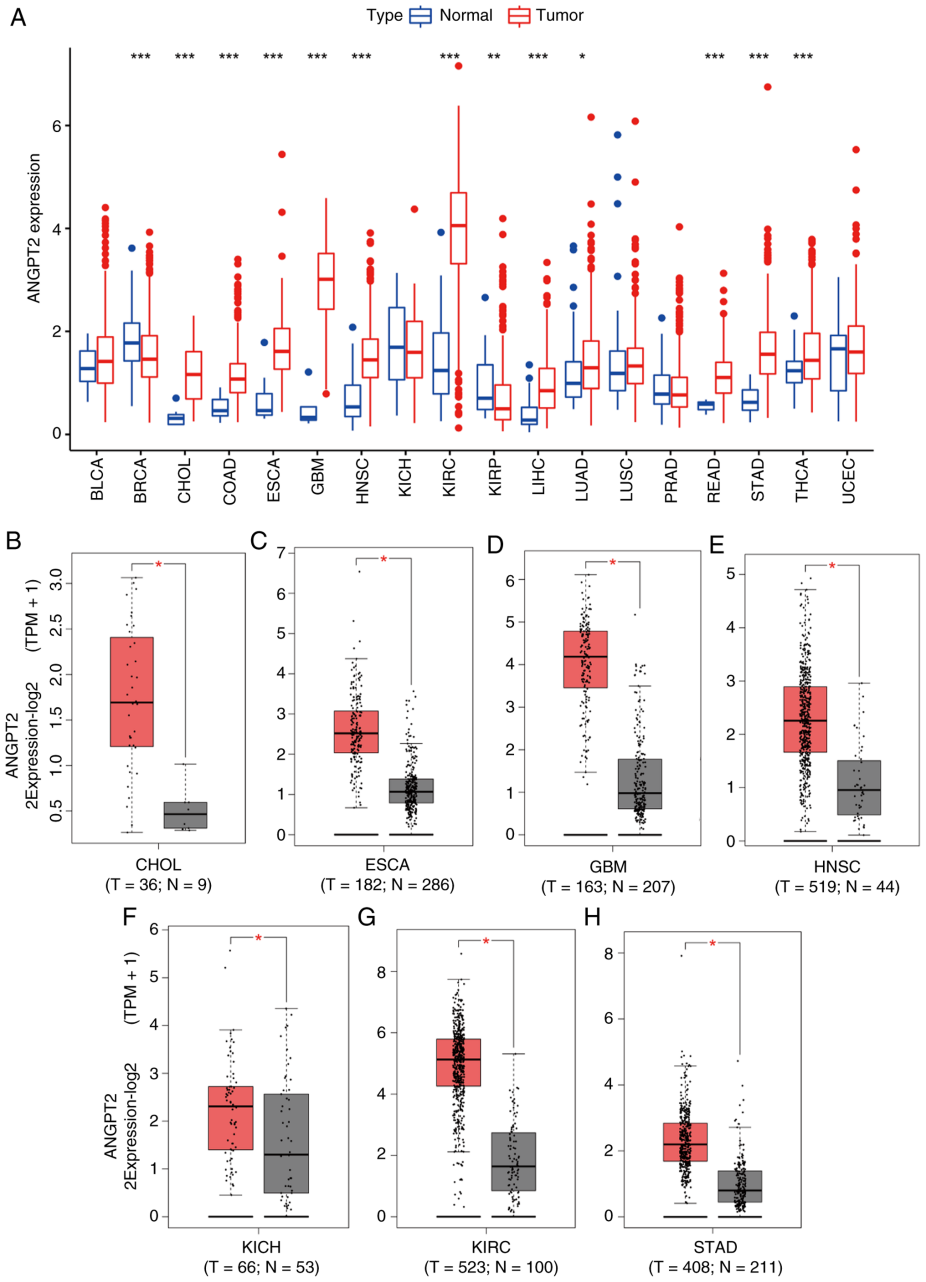
ANGPT2 expression in multiple tumors. (A) ANGPT2 levels in 18 human tumors using TCGA tumor and normal data. (B-H) ANGPT2 levels in TCGA; (B) CHOL, (C) ESCA, (D) GBM, (E) HNSC, (F) KICH, (G) KIRC and (H) STAD tissues vs. the matching TCGA and GTEx normal tissues. *P<0.05, **P<0.01 and ***P<0.001; all P-values are adjusted for multiple comparisons where applicable. ANGPT2, angiopoietin-2; TCGA, The Cancer Genome Atlas; CHOL, cholangiocarcinoma; ESCA, esophageal cancer; GBM, glioblastoma multiforme; HNSC, head and neck squamous cell carcinoma; KICH, kidney chromophobe; KIRC, kidney renal clear cell carcinoma; STAD, stomach adenocarcinoma.

Using the TCGA pan-cancer transcriptomic dataset, the analysis illustrated that ANGPT2 was significantly upregulated in 13 tumor types: BRCA, CHOL, COAD, ESCA, GBM, HNSC, LUAD, KIRC, KIRP, LIHC, READ, STAD and THCA, compared with normal tissues (Fig. 1A). No significant differential expression was detected in BLCA, KICH, LUSC, PRAD and UCEC tumors. Validation via the GEPIA platform further confirmed elevated ANGPT2 expression in CHOL, ESCA, HNSC, GBM, KICH, KIRC and STAD relative to normal tissues (Fig. 1B-H). ANGPT2 was upregulated in CHOL, ESCA, HNSC, GBM, KIRC and STAD, implicating it as a potential driver of oncogenesis and disease progression in these contexts.

### Prognostic analysis of ANGPT2 in tumors

**Figure 2.**
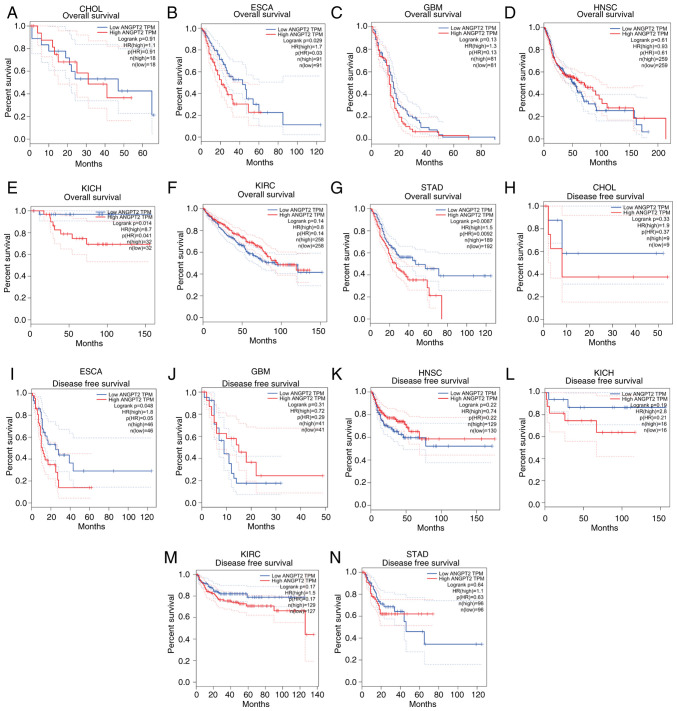
Gene expression profiling interactive analysis database: OS and DFS analysis of ANGPT2 in diverse human tumors. (A-G) OS plot: ANGPT2 in (A) CHOL, (B) ESCA, (C) GBM, (D) HNSC, (E) KICH, (F) KIRC and (G) STAD. (H-N) DFS plot: ANGPT2 in (H) CHOL, (I) ESCA, (J) GBM, (K) HNSC, (L) KICH, (M) KIRC and (N) STAD. OS, Overall survival; DFS, disease-free survival; ANGPT2, angiopoietin-2; CHOL, cholangiocarcinoma; ESCA, esophageal cancer; GBM, glioblastoma multiforme; HNSC, head and neck squamous cell carcinoma; KICH, kidney chromophobe; KIRC, kidney renal clear cell carcinoma; STAD, stomach adenocarcinoma.

Through the GEPIA database, the ANGPT2 prognostic value was ascertained in CHOL, ESCA, HNSC, GBM, KIRC and STAD, assessing both OS and DFS, revealing that overexpressed ANGPT2 was significantly linked to worse OS in ESCA and STAD (Fig. 2A-G). By contrast, for DFS, high ANGPT2 expression predicted adverse outcomes exclusively in ESCA (Fig. 2H-N). Further analysis of TCGA clinical data (Table I) confirmed a significant link between ANGPT2 levels and survival outcomes, specifically in ESCA. These findings suggest that ANGPT2, particularly when combined with OS and DFS metrics, serves as a robust biomarker of poor prognosis in patients with ESCA.

### Patient characteristics

To assess ANGPT2 expression in ESCA, clinical and transcriptomic data were retrieved from 183 patients with ESCA in the TCGA database, which included their demographics and clinicopathological characteristics, as shown in Table II.

### Correlation between clinical analysis and ANGPT2 expression

**Figure 3.**
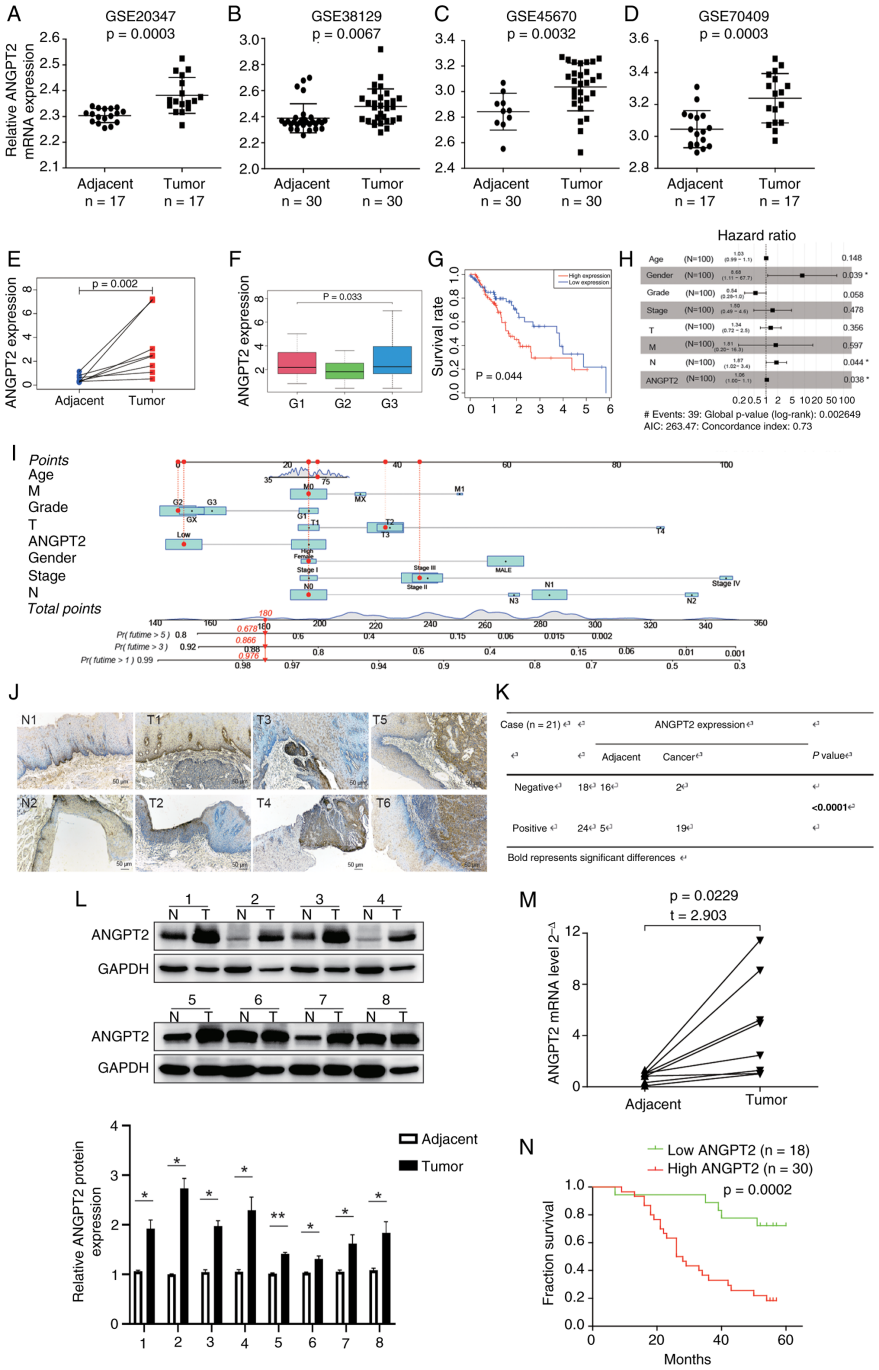
Association of ANGPT2 expression with clinical analysis in ESCA. (A-D) ANGPT2 expression in the GSE20347, GSE38129, GSE45670 and GSE70409 datasets. (E) Paired ANGPT2 expression in adjacent and tumor tissues. (F) Correlation between ANGPT2 expression and grade. (G) Impact of ANGPT2 expression on OS in patients with ESCA. (H) Multivariate Cox regression: Connection with OS and clinicopathologic characteristics in patients with ESCA. (I) Nomogram for the 1-, 3-, and 5-yr OS prediction of patients with ESCA. (J) Representative images and (K) statistical analysis: ANGPT2 expression in ESCA tumors and normal tissues. Scale bars, 50 µm. (K) Number of ANGPT2 expression negative or positive cases in normal tissues and ESCC tissues based on the IHC staining score results. The results demonstrated that 16 cases were negative and only 5 cases were positive for ANGPT2 expression in normal tissues, while merely 2 cases were negative and 19 cases were positive for ANGPT2 expression in ESCC tissues. (L) ANGPT2 protein expression in paired tissues obtained from patients with ESCA. (M) ANGPT2 mRNA expression levels in ESCA and paired adjacent normal tissues. (N) Kaplan-Meier: Prognosis of OS according to the IHC scores of ANGPT2. Statistical analyses: Paired Student's t-test was used for comparisons between paired tumor and normal tissues (E, L and M); unpaired Student's t-test was used for comparison of IHC scores between tumor and normal tissues (K); one-way ANOVA was used for analyzing ANGPT2 expression across different tumor grades (F); log-rank test was used for survival analyses (G and N); Cox proportional hazards model was used for multivariate regression analysis (H). *P<0.05 and **P<0.01; all P-values are adjusted for multiple comparisons where applicable. ANGPT2, angiopoietin-2; ESCA, esophageal cancer; OS, overall survival; ESCC, esophageal squamous cell carcinoma; IHC, immunohistochemical.

To evaluate ANGPT2 expression in ESCA, transcriptomic data from four independent GEO datasets (GSE20347, GSE38129, GSE45670 and GSE70409) were first analyzed. In all cohorts, ANGPT2 mRNA levels displayed significant overexpression in ESCA tissues relative to neighboring healthy tissues (GSE20347: P=0.0003; GSE38129: P=0.0067; GSE45670: P=0.0032; GSE70409: P=0.0003; Fig. 3A-D). Afterwards, the TCGA database was utilized to analyze 171 ESCA samples, resulting in the discovery of ANGPT2 expression data for every individual. According to the paired t-test, the ANGPT2 expression level in tumors was elevated compared with healthy tissues (P=0.002; Fig. 3E). The outcomes illustrated a significant relationship between ANGPT2 levels and the histological tumor grade (P=0.033; Fig. 3F). The survival analysis indicated that ESCA with elevated levels of ANGPT2 had a more unfavorable prognosis compared with ESCA with lower levels of ANGPT2 (P=0.044; Fig. 3G). In the analysis that considered multiple variables, ANGPT2 continued to be linked to OS independently, with a hazard ratio (HR) of 1.06 (Confidence interval: 1.00–1.1; P=0.038), in addition to lymph node status and sex (Fig. 3H). Next, a nomogram that included ANGPT2 expression and clinicopathological factors was developed for predicting the survival rates at 1, 3, and 5 years (Fig. 3I). IHC analyses of ANGPT2 protein levels in ESCA tumors and normal tissues revealed that ANGPT2 expression was significantly higher in cancerous tissues (Fig. 3J and K). Furthermore, IHC-based analysis further linked elevated ANGPT2 protein levels to lymph node metastasis and advanced histological grade (Table III). Concordantly, ANGPT2 mRNA and protein expression levels were consistently and significantly upregulated in ESCA tumors relative to normal tissues (Fig. 3L and M). To investigate whether ANGPT2 has prognostic value in patients with ESCA, data from The Affiliated Hospital of Jiangsu University (Zhenjiang, China) were utilized. The findings indicated that individuals diagnosed with ESCA who had elevated levels of ANGPT2 exhibited a poorer prognosis (P=0.0002; Fig. 3N).

### In vitro, ANGPT2 modulates migration and invasion of TE-1 and KYSE150 cells

The results revealed a significant increase in ANGPT2 levels in KYSE30, KYSE150 and TE-1 cells, unlike Het-1A cells (Fig. S1A). To examine the biological role of ANGPT2 in a laboratory setting, TE-1 cells and KYSE150 cells were subjected to si-ANGPT2 to suppress ANGPT2. Among the three ANGPT2-targeting siRNAs transfected into TE-1 and KYSE150 cells, two exhibited significantly higher knockdown efficacy compared with the non-targeting si-NC group. Notably, si-ANGPT2-3 achieved the most robust silencing effect, with an interference efficiency exceeding 70% at protein levels (Fig. S1B). The si-ANGPT2 group (Fig. S1C) exhibited a significant reduction in cells' migratory and invasive abilities, as demonstrated by Transwell assays. By contrast, the si-ANG2 group exhibited a reduction in cell migration during wound healing analysis compared with the control (Fig. S1D). The outcomes demonstrated that ANGPT2 had a notable impact on diminishing the movement and infiltration of ESCC cells.

### Forecasting and examination of ANGPT2′s upstream regulatory elements

**Figure 4.**
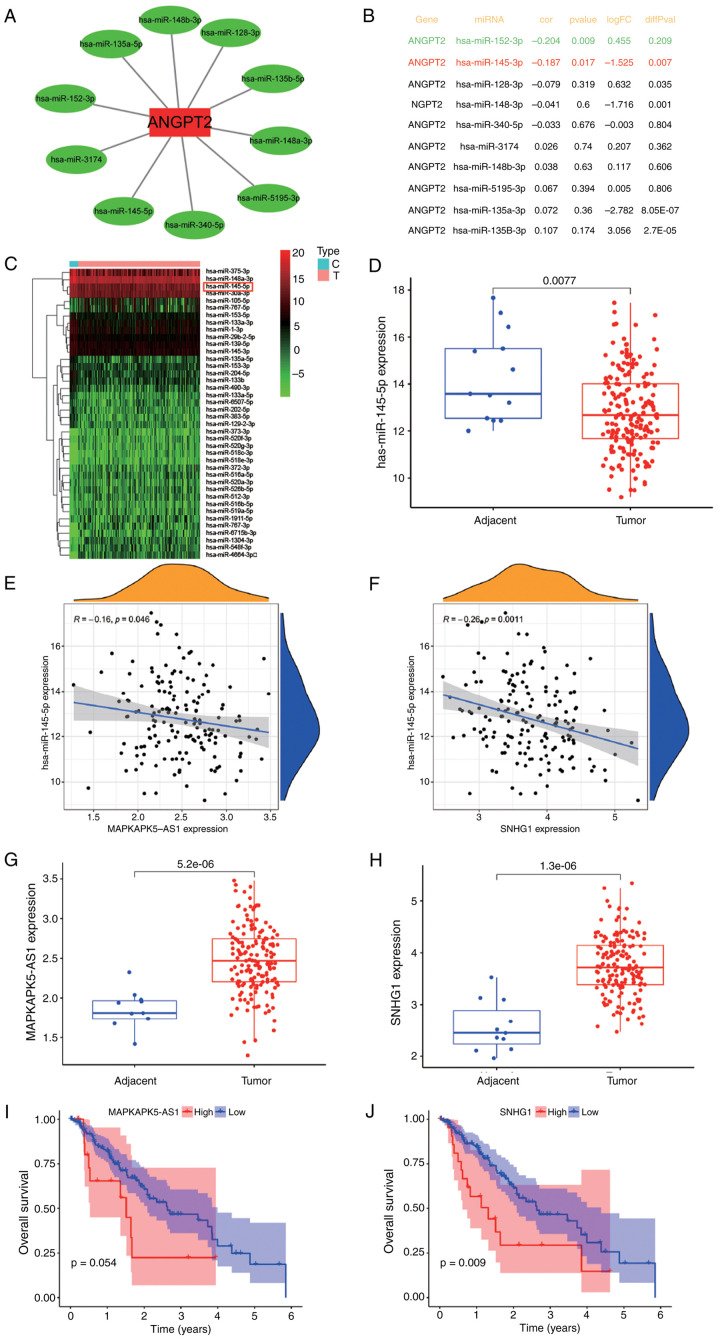
Upstream regulator of ANGPT2 in ESCA. (A) Cytoscape software: miRNA-ANGPT2 regulatory network. (B) Expression correlation between predicted miRNAs and ANGPT2 in ESCA. (C) Unsupervised hierarchical clustering heatmap based on the differentially expressed miRNAs between 185 ESCA tissues and 13 healthy tissues. (D) miR-145-5p level in ESCA and control normal samples. (E) Collation analysis: miR-145-5p and AMPKAPK5-AS1, as well as (F) miR-145-5p and SNHG1. (G) AMPKAPK5-AS1 and (H) SNHG1 expression levels in adjacent and tumor tissues. (I) Overall survival analysis: AMPKAPK5-AS1 and (J) SNHG1 in patients with ESCA. Statistical analyses: Unpaired Student's t-test was used for comparing lncRNA/miRNA expression levels between tumor and normal tissues (D, G and H); Spearman's rank correlation coefficient was used for evaluating correlations between gene expression levels (B, E and F); log-rank test was used for survival analyses (I and J). All P-values were adjusted for multiple comparisons using the Benjamini-Hochberg method to control the false discovery rate. All P-values are adjusted for multiple comparisons where applicable. ANGPT2, angiopoietin-2; ESCA, esophageal cancer; miRNAs or miRs, microRNAs.

**Figure 5.**
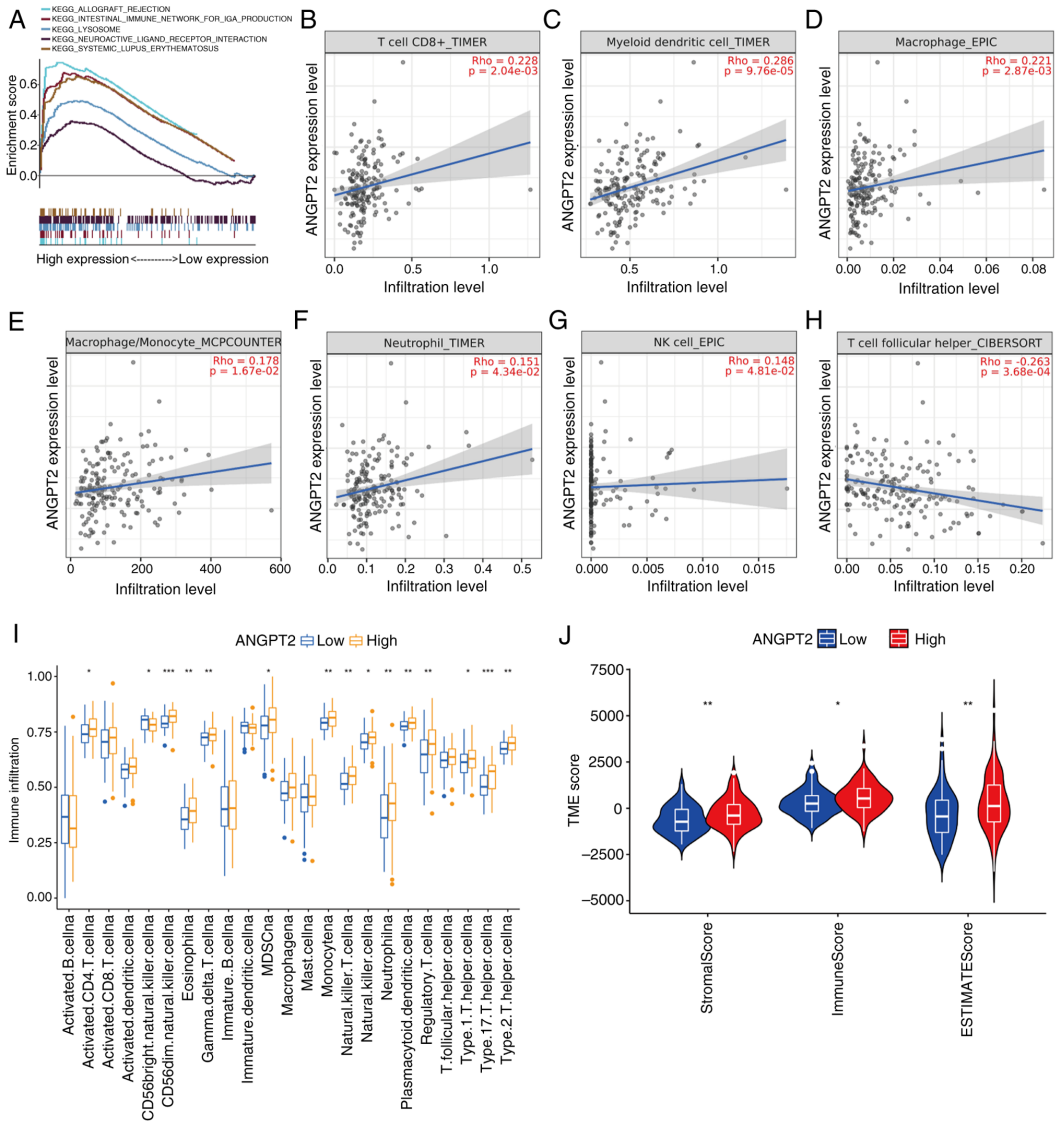
Connection of ANGPT2 expression with immunity in ESCA. (A) GSEA enrichment plots showing signaling pathways significantly enriched in the ANGPT2 high-expression group (1,000 permutations were performed; pathways include ‘ALLOGRAFT_REJECTION’ and ‘INTERFERON_GAMMA_RESPONSE’, consistent with immune-related functions). (B-H) Spearman's correlation analysis between ANGPT2 expression and infiltration levels of specific immune cell subsets in ESCA (adjusted for tumor purity via TIMER2.0 or indicated algorithms): (B) CD8^+^ T cells (TIMER, Rho=0.220, P=2.04×10^−3^); (C) myeloid dendritic cells (TIMER, Rho=0.286, P=9.76×10^−5^); (D) macrophages (EPIC, Rho=0.211, P=2.87×10^−3^); (E) macrophage/monocytes (MCPCOUNTER, Rho=0.178, P=1.67×10^−2^), (F) neutrophils (TIMER, Rho=0.132, P=4.34×10^−2^), (G) NK cells (EPIC, Rho=0.148, P=4.81×10^−2^), (H) T follicular helper cells (CIBERSORT, Rho=0.260, P=3.68×10^−4^). (I) Single-sample GSEA showing the abundance of 23 infiltrating immune cell subgroups in ANGPT2 high- vs. low-expression groups. (J) ESTIMATE algorithm analysis of Stromal Score, Immune Score and ESTIMATE Score in ANGPT2 high- vs. low-expression groups (P<0.01 for Stromal Score; P<0.05 for Immune Score; P<0.01 for ESTIMATE Score, as indicated by symbols). *P<0.05, **P<0.01 and ***P<0.001; all P-values are adjusted for multiple comparisons where applicable. ANGPT2, angiopoietin-2; ESCA, esophageal cancer; GSEA, Gene Set Enrichment Analysis; NK, natural killer.

The non-coding RNAs are well-established modulators of gene expression. To identify potential upstream regulators of ANGPT2, StarBase was employed to predict miRNAs with binding potential to the ANGPT2 3′ untranslated region (3′ UTR), requiring consensus across at least two prediction algorithms. This yielded 10 candidate miRNAs, which were visualized in a regulatory network using Cytoscape (v3.9.1; http://cytoscape.org/). (Fig. 4A). Given that miRNAs exert regulatory control by suppressing their target genes (20), an inverse correlation between candidate miRNAs and ANGPT2 was hypothesized. Correlation analysis using TCGA-ESCA data revealed that only miR-152-3p and miR-145-5p exhibited significant negative correlations with ANGPT2 expression (Fig. 4B). Of these, only miR-145-5p was significantly impaired in ESCA tissues (P=0.0077; Fig. 4C and D), positioning it as the most plausible direct regulator of ANGPT2 in ESCA. StarBase was then utilized to forecast the upstream lncRNA of miR-145-5p, to identify the regulatory factors influencing its expression. According to the present results, 38 kinds of lncRNAs could bind miRNAs. miRNAs can induce gene silencing through their binding to mRNAs, while IncRNAs can regulate gene expression through their competitive binding to miRNAs. IncRNAs can bind to miRNAs through miRNA response elements, thereby impacting gene silencing caused by miRNAs. Hence, an inverse relationship is expected to exist between lncRNAs and miRNAs. Through the TCGA analysis (Fig. 4E and F), only MAPKAPK5-AS1 (P=0.046) and SNHG1 (P=0.0011) displayed a negative link with miR-145-5p. Eventually, the significance and predictive capabilities of MAPKAPK5-AS1 and SNHG1 were explored in ESCA. As demonstrated in Fig. 5G-J, MAPKAPK5-AS1 was significantly upregulated in ESCA (P=5.2×10^−6^), but its upregulation did not have a significant influence on patient prognosis (P=0.054). SNHG1 was also significantly upregulated in ESCA (P=1.3×10^−6^), and SNHG1 overexpression in ESCA suggested that patients had a poor prognosis (P=0.009). Combined with the aforementioned analysis, MAPKAPK5-AS1 and SNHG1 may negatively regulate miR-145-5p and promote ANGPT2 expression. To validate this regulatory axis (MAPKAPK5-AS1/SNHG1 → miR-145-5p → ANGPT2), dual-luciferase reporter assays were performed. WT and MUT recombinant vectors of ANGPT2 3′UTR, MAPKAPK5-AS1 and SNHG1 were constructed, with the binding region sequence mutated from ‘AACTGGA’ to ‘TTGACCT’ (Fig. S3A-C). The dual-luciferase reporter assay results demonstrated that miR-145-5p mimic significantly reduced the luciferase activity of the ANGPT2 3′UTR-WT vector, but had no obvious effect on the MUT vector; meanwhile, both MAPKAPK5-AS1-WT and SNHG1-WT vectors could significantly inhibit the inhibitory effect of miR-145-5p mimic on luciferase activity, while the MUT vectors lost this regulatory effect (Fig. S3D-F). These findings confirmed the direct binding of miR-145-5p to the 3′UTR of ANGPT2, and the specific binding of MAPKAPK5-AS1 and SNHG1 to miR-145-5p, verifying the existence of the MAPKAPK5-AS1/SNHG1/miR-145-5p/ANGPT2 ceRNA regulatory axis in ESCA.

### Connection between ANGPT2 expression and immunity

To ascertain the differentially activated signaling pathways in ESCA, a GSEA was conducted on the datasets of ANGPT2 with high and low expression. The signaling pathway with the most significant enrichment was selected based on its NES. The ANGPT2 high-expression phenotype was differentially enriched in allogeneic rejection, IGA-producing intestinal immune network, neuroactive ligand receptor interaction, lysosome and systemic lupus erythematosus (SLE) (Fig. 5A). These enrichment results suggested that ANGPT2 was related to immunity. The ICs infiltrating tumors can profoundly affect tumor progression. The correlation between ANGPT2 expression in ESCA and the analyzed ICs, such as CD8^+^ T cells, dendritic cells (DCs), monocytes, macrophages (MPh), neutrophils, natural killer cells (NKs) and T cell follicular helper cells, was significant (Fig. 5B-H). To quantify differences in immune infiltration across the broader immune landscape, ssGSEA was used to compare 23 distinct IC subsets between high- and low-ANGPT2 groups. Results demonstrated significant disparities in immune infiltration profiles, with high-ANGPT2 tumors exhibiting elevated infiltration across multiple IC subtypes (Fig. 5I). Further characterization of the TME using the ESTIMATE algorithm manifested that ANGPT2 overexpression correlated with significantly higher stromal, immune and ESTIMATE scores (Fig. 5J), indicating a more immune- and stroma-rich TME, consistent with enhanced immune recruitment or remodeling. To probe the mechanistic link between ANGPT2 and immune activation, correlations between ANGPT2 levels and established IC marker genes in ESCA were analyzed using TCGA data (Table IV). ANGPT2 showed strong positive correlations with markers of T cell (CD3G and CD4), T follicular helper cell (ICOS), M1-MPh (NOS2, IRF5 and PTGS2), M2-MPh (VSIG4, CD163 and MS4A4A), neutrophil cell (FCGR3B), DC (HLA-DPB1, HLA-DRA, NRP1, HLA-DPA1 and ITGAX), NKs (NKG7, FCGR3A) and monocyte cell (S100A8, S100A9, CD68 and LYZ). ANGPT2 is highly correlated with immune invasion, but its expression in ICs is very low. The results of single-cell sequencing of ESCA are depicted in Fig. S2. The aforementioned results partially support that ANGPT2 is positively correlated with immunity.

### Relationship between ANGPT2 and immune checkpoints and chemotherapeutic sensitivity in ESCA

**Figure 6.**
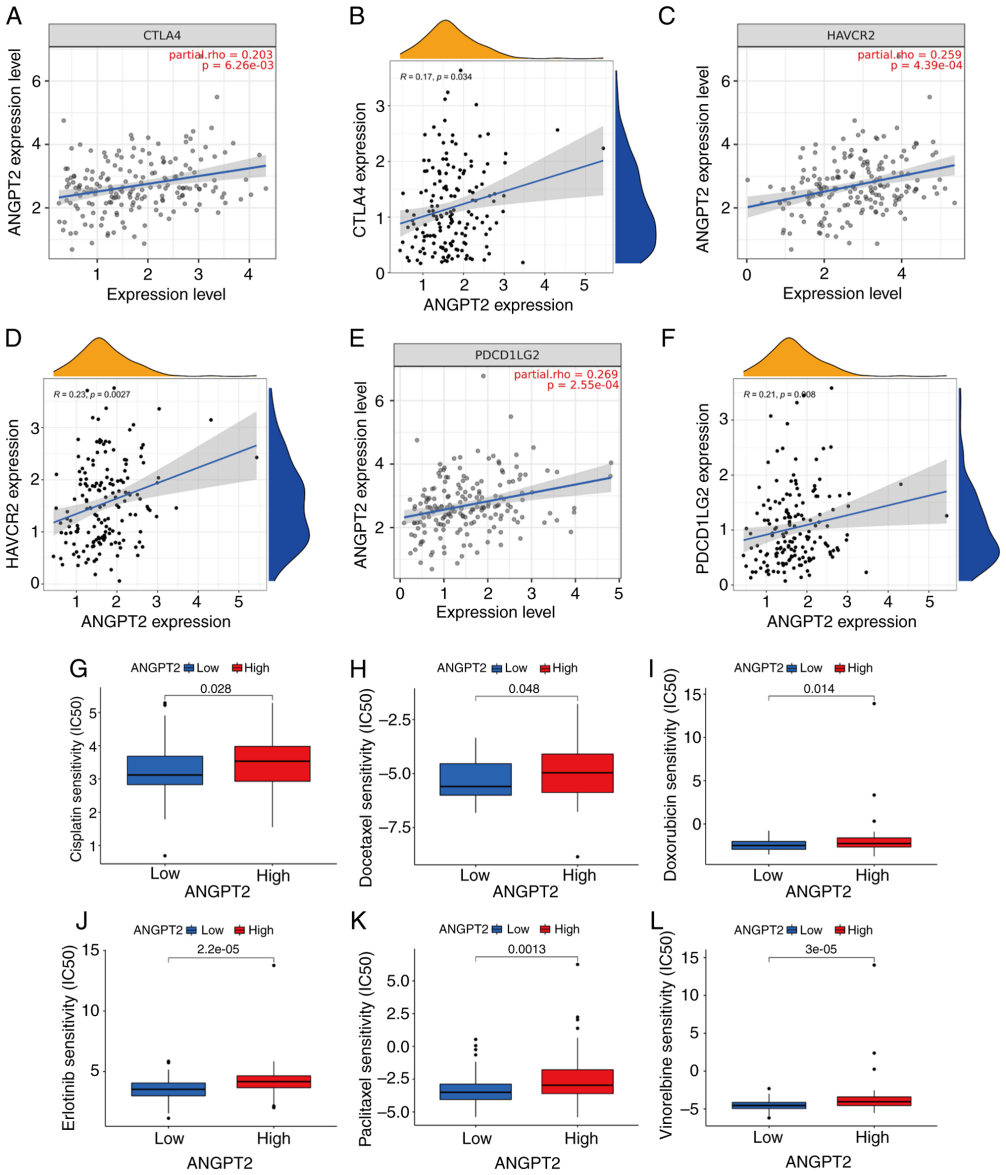
Relation of ANGPT2 level with immune checkpoint and chemotherapeutic sensitivity in ESCA. (A) TIMER-conducted Spearman correlation: ANGPT2 with CTLA-4 expression in ESCA. (B) TCGA-determined expression correlation: ANGPT2 with CTLA4 in ESCA. (C) TIMER-conducted Spearman correlation: ANGPT2 with HAVCR2 expression in ESCA. (D) TCGA-determined expression correlation: ANGPT2 with HAVCR2 in ESCA. (E) TIMER-conducted Spearman correlation: ANGPT2 with PDCD1LG2 expression in ESCA. All TIMER-conducted Spearman correlations were adjusted by purity. (F) TCGA-determined expression correlation: ANGPT2 with PDCD1LG2 in ESCA. (G-L) Relationships between both ANGPT2 expression groups and chemotherapeutic sensitivity. ANGPT2, angiopoietin-2; ESCA, esophageal cancer; TCGA, The Cancer Genome Atlas.

Tumor cells can modify the TME and facilitate immune system evasion by the tumor (21). The relation between 70 immune checkpoints and ANGPT2 was analyzed via the TCGA database (Table SI; Fig. 6B, D and F). Immune checkpoints, including CTLA-4 (22), HAVCR2 (23) and PDCD1LG2 (24), play a key role in cancer progression. Since ANGPT2 may play a carcinogenic role in ESCA, the relationship between ANGPT2 and CTLA-4, as well as HAVCR2 and PDCD1LG2, was also evaluated using TIMER2.0. ANGPT2 expression in ESCA was positively related to CTLA-4, HAVCR2 and PDCD1LG2 (Fig. 6A, C and D). The findings suggest that the evasion of the immune system by tumors may be involved in ESCA development through the action of ANGPT2. To explore its potential clinical relevance in therapeutic response, the sensitivity of ESCA tumors stratified by ANGPT2 expression levels to commonly used chemotherapeutic agents, was evaluated. Notably, the low ANGPT2 expression group exhibited lower IC_50_ values for chemotherapeutics, including cisplatin, docetaxel, doxorubicin, erlotinib, paclitaxel and vinorelbine. To summarize, the findings suggest a correlation between drug sensitivity and ANGPT2 (Fig. 6G-L).

## Discussion

To date, despite the comprehensive treatment available for ESCA, the prognosis is still unsatisfactory. Exploring the molecular mechanism of ESCA carcinogenesis and identifying promising prognostic biomarkers are extremely critical. ANGPT2 has been reported to be crucial not only in CVD but also in initiation and progression of human tumors. However, its role in ESCA remains underexplored, necessitating further mechanistic and clinical investigation.

The current study conducted a pan-cancer analysis of ANGPT2 expression through the TCGA database, with validation performed via the GEPIA. Survival analysis showcased that patients with ESCA with overexpressed ANGPT2 exhibited significantly worse OS. Multivariate clinical data analysis further indicated that ANGPT2 overexpression may serve as an independent prognostic factor in ESCA. *In vitro* functional assays demonstrated that knocking down ANGPT2 significantly suppressed ESCA migration and invasion. Collectively, ANGPT2 plays an oncogenic role in ESCA progression. ANGPT2 exhibits significant stage-dependent overexpression in ESCA. In TCGA and GEO cohorts, its mRNA levels escalate with TNM stages (I–IV), showing marked differences between adjacent stages (all P<0.05). Local IHC data confirm higher protein scores in advanced (III–IV, median=7.8) vs. early stages (I–II, median=3.2, P<0.001), correlating with T/N/M classifications. ANGPT2 distinguishes early from advanced ESCA with area under the curves (AUCs) of 0.78 (mRNA) and 0.86 (protein), improving to 0.92 when combined with CEA/SCC. It also aids early diagnosis, with AUCs of 0.73 (mRNA) and 0.80 (protein) vs. normal tissues. Mechanistically, stage-dependent upregulation via the MAPKAPK5-AS1/SNHG1/hsa-miR-145-5p axis drives angiogenesis, TME remodeling and immune checkpoint (CTLA-4, HAVCR2 and PDCD1LG2) activation. Clinically, it refines TNM staging (C-index=0.78) and enables non-invasive monitoring via serum levels (AUC=0.83). These data support ANGPT2 as a valuable diagnostic biomarker for ESCA stage stratification and early detection.

miRNAs and lncRNAs are key regulators of gene expression. To identify potential upstream miRNAs targeting ANGPT2, StarBase was used to predict miRNA-ANGPT2 interactions. Finally, 10 miRNAs were obtained, and some of the predicted miRNAs had been verified to have a tumor suppressor role in human tumors. For instance, hsa-miR-148a-3p is associated with drug resistance and aggressiveness in ESCA (20), and miR-152 functions as a tumor suppressor in human BRCA by targeting PIK3CA (21). After correlation and expression analyses, hsa-miR-145-5p was chosen as the most promising upstream tumor suppressor miRNA of ANGPT2. High hsa-miR-145-5p expression in ESCA is related to prognosis (22). In addition, lncRNAs potentially interacting with hsa-miR-145-5p were predicted, identifying 38 candidate lncRNAs. Through correlation and expression analyses, as well as survival prediction analysis, two lncRNAs, MAPKAPK5-AS1 and SNHG1, were prioritized. Both have been implicated in the pathogenesis of multiple malignancies. For instance, MAPKAPK5-AS1 drives LIHC progression via a MAPKAPK5-AS1/PLAGL2/HIF-1α signaling axis (23), while SNHG1 knockdown in ESCA suppresses migration and invasion and promotes apoptosis through miR-204 upregulation and HOXC8 downregulation (24). In summary, the MAPKAPK5-AS1 and SNHG1/hsa-miR-145-5p/ANGPT2 axes may represent novel regulatory signaling contributing to ESCA pathogenesis. The dual-luciferase reporter assay results have confirmed the direct binding between the predicted lncRNAs (MAPKAPK5-AS1 and SNHG1) and miR-145-5p, as well as between miR-145-5p and the 3′UTR of ANGPT2, providing experimental evidence for the post-transcriptional regulatory mechanism of this axis. However, RNA immunoprecipitation assays to verify the endogenous physical interaction between these lncRNAs and miR-145-5p in ESCA cells have not been performed in the current study; this experiment is planned for follow-up research to fully characterize the competing endogenous RNA regulatory network in ESCA and confirm the *in vivo* functional relevance of this axis.

Emerging evidence indicates that overexpressed ANGPT2 is associated with increased all-cause and cardiovascular mortality in the general population, and increased death in patients with cardiogenic shock (25,26). ANGPT2 levels are highly expressed in human malignancies and CVD, including heart failure, ischemic myocardial injury, and other complications secondary to chronic kidney impairment, diabetes and hypertension. Despite distinct clinical phenotypes, cancer and CVD share key ANGPT2-driven pathogenic mechanisms, particularly poor vascular network remodeling, inflammation and epithelial (or endothelial) to mesenchymal transition. ANGPT2-dependent vascular instability is a well-known underlying mechanism behind malignancy and CVD progression (27). In addition, ANGPT2 has been shown to exert immunomodulatory effects by inducing PD-L1 overexpression in tumor-associated MPh, thereby impairing T-cell-mediated immune surveillance and antitumor cytotoxicity. These findings position ANGPT2 not only as a functional contributor to immune evasion but also as a probable biomarker and therapeutic target within the immune checkpoint landscape (28). Studies have shown that endothelial cell- and MPh-derived Angpt2 aggravates cardiac hypoxia and inflammation by promoting abnormal vascular remodeling, enhancing neutrophil infiltration, and pro-inflammatory MPh polarization after myocardial ischemia and myocardial infarction (29).

In the present study, the phenotype of ANGPT2 overexpression is correlated with allogeneic rejection, neuroactive ligand receptor interaction, the IGA-producing intestinal immune network, lysosome function and SLE, as determined by GSEA, all of which underscore its immunological relevance. Given the accumulating evidence that immune dysregulation contributes to tumorigenesis, the findings of the present study further demonstrate that ANGPT2 has a high correlation with ICs, including CD8^+^ T cells, MPh, DCs, monocytes, neutrophils, NK cells and T cell follicular helper cells. Moreover, the TME attributes and the relative abundance of 23 TIICs differed significantly between ANGPT2 expression groups. Additionally, ANGPT2 overexpression in ESCA is closely related to the immune checkpoints CLAT-4, HAVCR2 and PDCD1LG2, indicating the implication of tumor immunity in the carcinogenesis of ESCA mediated by ANGPT2. Moreover, ANGPT2 was related to drug sensitivity.

Notably, the current findings on the association between ANGPT2 and tumor immunity provide a strong rationale for exploring its potential value in ESCA immunotherapy, especially in combination with PD-1/PD-L1 inhibitors. Immune checkpoint inhibitors (ICIs) targeting the PD-1/PD-L1 axis have transformed the treatment of various cancers, but their efficacy in ESCA remains limited, with only ~20–30% of patients achieving durable responses (30). This limited efficacy is largely attributed to the immunosuppressive TME, which restricts T cell infiltration and functional activation. The present data reveal that ANGPT2 overexpression is positively correlated with PDCD1LG2 (PD-L2) expression, a key ligand of PD-1, and the enrichment of immunosuppressive cells such as M2 macrophages and myeloid-derived suppressor cells MDSCs in ESCA. This aligns with previous findings demonstrating that ANGPT2 can induce PD-L1 upregulation on tumor-associated macrophages and tumor cells, thereby fostering immune evasion (19). Collectively, these observations suggest that ANGPT2 may be a critical driver of ICI resistance in ESCA, and targeting ANGPT2 could synergize with PD-1/PD-L1 blockade to enhance therapeutic efficacy.

Preclinical studies in other cancer types have already validated the synergistic effects of ANGPT2 inhibition and PD-1/PD-L1 inhibitors. For example, in non-small cell lung cancer, neutralizing ANGPT2 normalized tumor vasculature, increased intratumoral CD8^+^ T cell infiltration, and enhanced the antitumor activity of anti-PD-1 therapy (18). In hepatocellular carcinoma, ANGPT2 knockdown reversed the immunosuppressive TME by reducing regulatory T cell accumulation and restoring effector T cell function, thereby sensitizing tumors to PD-L1 inhibitors (31). Extrapolating these findings to ESCA, it was hypothesized that ANGPT2 inhibition could remodel the immunosuppressive TME characterized by high PDCD1LG2 expression and abnormal immune cell infiltration. Specifically, targeting ANGPT2 may downregulate PD-L1/PD-L2 expression on tumor cells and macrophages, reduce the recruitment of immunosuppressive cells, and enhance the cytotoxic activity of CD8^+^ T cells, ultimately overcoming ICI resistance and improving treatment outcomes.

The clinical implications of this combination strategy are substantial. Patients with ESCA with high ANGPT2 expression, who exhibit poor prognosis and compromised tumor immunity, may be ideal candidates for dual ANGPT2/PD-1/PD-L1 targeting. ANGPT2 could serve as a predictive biomarker to identify patients most likely to benefit from ICI-based combination therapy, addressing the unmet need for personalized immunotherapy in ESCA. Furthermore, the observation of the present study that ANGPT2 expression correlates with chemotherapeutic sensitivity suggests that triple therapy (ANGPT2 inhibition + PD-1/PD-L1 blockade + chemotherapy) may yield even greater clinical benefits. Chemotherapy can induce immunogenic cell death, release tumor-associated antigens, and synergize with immunotherapy to amplify antitumor immune responses (32), while ANGPT2 inhibition may further enhance this synergy by remodeling the TME.

However, several considerations must be addressed before clinical translation. First, the specific molecular mechanisms by which ANGPT2 regulates PD-L1/PD-L2 expression in ESCA require further validation, for example, whether ANGPT2 directly modulates the transcriptional activity of PDCD1LG2 or acts through downstream signaling pathways such as PI3K/Akt or NF-κB. Second, preclinical studies using ESCA cell line-derived xenografts and patient-derived xenografts (PDXs) are needed to confirm the synergistic efficacy of ANGPT2 inhibitors (for example, neutralizing antibodies and siRNA) and PD-1/PD-L1 blockers. Third, the optimal dosage, administration schedule and safety profile of ANGPT2-targeting agents in combination with ICIs must be evaluated in preclinical models to minimize toxicity while maximizing efficacy. Finally, clinical trials are warranted to assess the safety and efficacy of this combination strategy, with correlative studies to monitor TME remodeling (for example, CD8^+^ T cell infiltration and PD-L1 expression) and validate ANGPT2 as a predictive biomarker.

In addition to immunotherapy, the crosstalk between ANGPT2 and other oncogenic pathways in ESCA merits further exploration. For instance, ANGPT2 has been reported to interact with VEGFA to promote angiogenesis and immune suppression (19), and dual targeting of ANGPT2 and VEGFA has shown promising results in preclinical models (33). Whether this dual anti-angiogenic strategy can synergize with immunotherapy in ESCA deserves investigation. Furthermore, the current identification of the MAPKAPK5-AS1/SNHG1/hsa-miR-145-5p/ANGPT2 regulatory axis provides additional therapeutic targets. Targeting MAPKAPK5-AS1 or SNHG1 could downregulate ANGPT2 expression, offering an alternative approach to modulate ANGPT2 activity in ESCA. However, the potential off-target effects of lncRNA-targeting therapies need to be carefully evaluated, and more specific targeting strategies (for example, antisense oligonucleotides and small molecule inhibitors) should be developed.

It is also important to acknowledge the limitations of the present study. First, the *in vitro* functional assays were performed in only two ESCA cell lines (TE-1 and KYSE150), and validation in additional cell lines and *in vivo* models (for example, PDXs) is necessary to confirm the oncogenic role of ANGPT2 in ESCA. Second, the correlation between ANGPT2 expression and immune infiltration was based on bioinformatics analysis of public databases, and experimental validation using clinical samples (for example, flow cytometry and multiplex immunofluorescence) is required to confirm these findings. Third, the mechanism by which ANGPT2 regulates the TME and immune checkpoints in ESCA remains partially elusive, and further studies are needed to dissect the detailed signaling pathways. Finally, the drug sensitivity analysis was based on the pRRophetic algorithm, and experimental validation using ESCA cell lines and patient-derived organoids is needed to confirm the correlation between ANGPT2 expression and chemotherapeutic sensitivity.

In summary, ANGPT2 is overexpressed in multiple tumors, including ESCA, and functions as an independent prognostic biomarker. It was found that MAPKAPK5-AS1 and SNHG1/hsa-miR-145-5p can regulate ANGPT2. In addition, tumor immunity may be involved in ESCA carcinogenesis, which is modulated by ANGPT2. Notably, ANGPT2 holds significant potential as a therapeutic target for enhancing the efficacy of PD-1/PD-L1 inhibitors in ESCA, providing a rationale for combination immunotherapy strategies. While these results provide a compelling mechanistic and clinical framework, they require further validation through functional *in vitro* and *in vivo* studies, as well as prospective, large-scale, multicenter clinical trials, to confirm their translational relevance and therapeutic potential.

Finally, the present study has certain limitations that should be acknowledged. First, consistent with the focused research design centered on ANGPT2, the expression levels of the upstream regulatory molecules (MAPKAPK5-AS1, SNHG1 and miR-145-5p) were not validated in the clinical samples of patients with ESCA. Although the regulatory relationships among these molecules were confirmed by *in silico* analysis and *in vitro* functional assays, the clinical relevance and expression patterns of this lncRNA/miRNA axis in patient tissues remain to be elucidated. Future studies with expanded sample sizes are warranted to validate the expression of this regulatory axis and explore its clinical prognostic value in ESCA.

## Supplementary Material

Supporting Data

Supporting Data

## Figures and Tables

**Table I.** Relationship between differentially expressed genes and patient survival in esophageal cancer.

Gene	KM	HR	HR.95L	HR.95H	Cox P-value
GPER1	5.8×10^−4^	0.56	0.34	0.94	3.0×10^−2^
EFNA1	9.3×10^−4^	1.51	1.08	2.13	1.8×10^−2^
ABRACL	2.1×10^−3^	1.88	1.30	2.71	7.5×10^−4^
PIGU	5.5×10^−3^	1.88	1.06	3.31	3.0×10^−2^
MT1E	6.5×10^−3^	0.84	0.73	0.97	1.7×10^−2^
PGK1	9.2×10^−3^	1.81	1.12	2.91	1.4×10^−2^
LINC00365	1.0×10^−2^	1.62	1.06	2.50	1.5×10^−2^
SNRPB	1.5×10^−2^	1.72	1.11	2.67	1.4×10^−2^
HSPD1	1.6×10^−2^	1.74	1.22	2.48	2.3×10^−3^
ANGPT2	1.8×10^−2^	1.35	1.00	1.82	4.8×10^−2^
GLA	2.2×10^−2^	1.65	1.17	2.34	4.5×10^−3^
HSP90AB1	2.3×10^−2^	1.45	1.17	1.98	1.8×10^−2^
SPDL1	2.5×10^−2^	1.60	1.02	2.50	3.8×10^−2^
CACYBP	2.5×10^−2^	1.74	1.07	2.82	2.3×10^−2^
H1-4	2.8×10^−2^	1.40	1.06	1.87	1.9×10^−2^
PTGSE3	3.0×10^−2^	2.59	1.47	4.57	1.0×10^−3^
MRPL47	3.7×10^−2^	1.57	1.03	2.39	3.5×10^−2^

**Table II.** Clinical characteristics of patients with esophageal carcinoma from The Cancer Genome Atlas.

Clinical characteristics	Total (n=183)	Percentage, %
Age, years		
≤60	91	49.7
>60	92	50.3
Sex		
Female	27	14.8
Male	156	85.2
Grade		
G1	19	13.3
G2	76	53.1
G3	48	33.6
Stage		
I	18	11.3
II	78	48.7
III	55	34.4
IV	9	5.6
T		
T1	31	18.8
T2	43	26.1
T3	86	52.1
T4	5	3
N		
N0	76	46.6
N1	68	41.7
N2	12	7.4
N3	7	4.3
M		
M0	134	93.7
M1	9	6.3

**Table III.** Specimens assayed for ANGPT2 expression.

		Expression level of ANGPT2		
			
Clinicopathological characteristics	Total (n=48)	Low	High	P-value
Sex				0.1018
Male	31	9	22	
Female	17	9	8	
Age				0.8776
<60	18	7	11	
≥60	30	11	19	
Tumor location				0.8811
Upper/Middle	26	10	16	
Lower	22	8	14	
Differentiation				0.0214
Poor	13	3	10	
Moderately	11	8	3	
High	24	7	17	
TNM stage				0.0736
I–II	24	12	12	
III–IV	24	6	18	
Lymphatic metastasis				0.0110
No	26	14	12	
Yes	22	4	18	
Distant metastasis				0.0913
No	33	15	18	
Yes	15	3	12	
Recurrence				0.0538
No	35	16	19	
Yes	13	2	11	

ANGPT2, angiopoietin-2.

**Table IV.** Correlation analysis between angiopoietin-2 and immune cell biomarkers in esophageal cancer determined by The Cancer Genome Atlas.

Immune cell	Biomarker	Cor	P-value
B cell	CD19	0.01	8.8×10^−1^
	CD79A	−0.7	3.2×10^−1^
T cell	CD3D	0.13	8.3×10^−2^
	CD3G	0.18	1.9×10^−2^
	CD4	0.28	3.0×10^−4^
Tfh cell	ICOS	0.21	5.0×10^−3^
M1 macrophage	NOS2	0.18	1.7×10^−2^
	IRF5	−0.24	1.9×10^−3^
	PTGS2	0.20	7.4×10^−3^
M2 macrophage	CD163	0.30	1.0×10^−4^
	VSIG4	0.21	6.5×10^−3^
	MS4A4A	0.29	1.1×10^−4^
Neutrophil	FCGR3B	0.23	2.4×10^−3^
	CXCR2	−0.09	2.5×10^−1^
Dendritic cell	HLA-DPB1	0.22	4.8×10^−3^
	HLA-DQB1	0.15	4.5×10^−2^
	HLA-DRA	0.25	1.2×10^−3^
	HLA-DPA1	0.26	6.4×10^−4^
	CD1C	−0.05	5.2×10^−1^
	NRP1	0.28	2.1×10^−4^
	ITGAX	0.26	6.2×10^−4^
Natural killer cell	NKG7	0.04	5.3×10^−1^
	FCGR3A	0.23	2.3×10^−3^
Monocyte cell	S100A8	−0.28	2.1×10^−4^
	S100A9	−0.27	3.4×10^−4^
	CD68	0.16	3.6×10^−2^
	LYZ	0.35	5.1×10^−6^

## Data Availability

The data generated in the present study may be found in the Gene Expression Omnibus under accession numbers GSE20347, GSE38129, GSE45670, GSE70409 and GSE160269 or at the following URL: https://www.ncbi.nlm.nih.gov/geo/query/acc.cgi?acc=GSE20347; https://www.ncbi.nlm.nih.gov/geo/query/acc.cgi?acc=GSE38129; https://www.ncbi.nlm.nih.gov/geo/query/acc.cgi?acc=GSE45670; https://www.ncbi.nlm.nih.gov/geo/query/acc.cgi?acc=GSE70409; https://www.ncbi.nlm.nih.gov/geo/query/acc.cgi?acc=GSE160269. The data generated in the present study may be requested from the corresponding author.

## Abbreviations

BLCA: bladder urothelial carcinoma
BRCA: breast invasive carcinoma
CHOL: cholangiocarcinoma
COAD: colon adenocarcinoma
GBM: glioblastoma multiforme
HNSC: head and neck squamous cell carcinoma
KICH: kidney chromophobe
KIRC: kidney renal clear cell carcinoma
KIRP: kidney renal papillary cell carcinoma
LIHC: liver hepatocellular carcinoma
LUAD: lung adenocarcinoma
LUSC: lung squamous cell carcinoma
PRAD: pancreatic adenocarcinoma
READ: rectum adenocarcinoma
STAD: stomach adenocarcinoma
THCA: thyroid carcinoma
UCEC: uterine corpus endometrial carcinoma
