# Supplementary material for: Unveiling the role of ANGPT2 in esophageal cancer: A prognostic factor and potential oncogene
Source: Oncol Rep. 2026 Mar 12;55(5):93. doi: 10.3892/or.2026.9098 (PMC13007277; doi:10.3892/or.2026.9098)

Figure S1. Knockdown of ANGPT2 inhibits migration and invasion of ESCA cells. (A) Western blot analysis of ANGPT2 protein expression in the immortalized normal esophageal squamous epithelial cell line (Het-1A) and three ESCA cell lines (KYSE30, KYSE150 and TE-1), with GAPDH as the internal reference. (B) Western blotting verification of ANGPT2 knockdown efficiency in TE-1 and KYSE150 cells after transfection with three ANGPT2-targeting siRNAs (si-ANGPT2-1/2/3) or non-targeting negative control siRNA (si-NC). Group definitions: si-NC=cells transfected with negative control siRNA; si-ANGPT2-1/2/3=cells transfected with ANGPT2-specific siRNAs. (C) Transwell migration and invasion assays of TE-1 and KYSE150 cells after ANGPT2 knockdown (si-ANGPT2-3, selected for highest knockdown efficiency) or si-NC treatment. Experimental conditions: Migration assays (24 h incubation, serum-free medium in top chamber, 10% FBS in bottom chamber); invasion assays (48 h incubation, Matrigel-precoated membrane). Scale bars, 50 μm. (D) Wound healing: Cells were scratched with a 200-μl pipette tip at 80-85% confluence, cultured in serum-free medium for 24 h, and images were captured at 0 and 24 h. Scale bars, 50 μm. *P<0.05, **P<0.01 and ***P<0.001. ANGPT2, angiopoietin-2; ESCA, esophageal cancer; siRNA, small interfering RNA; NC, negative control; ns, not significant.

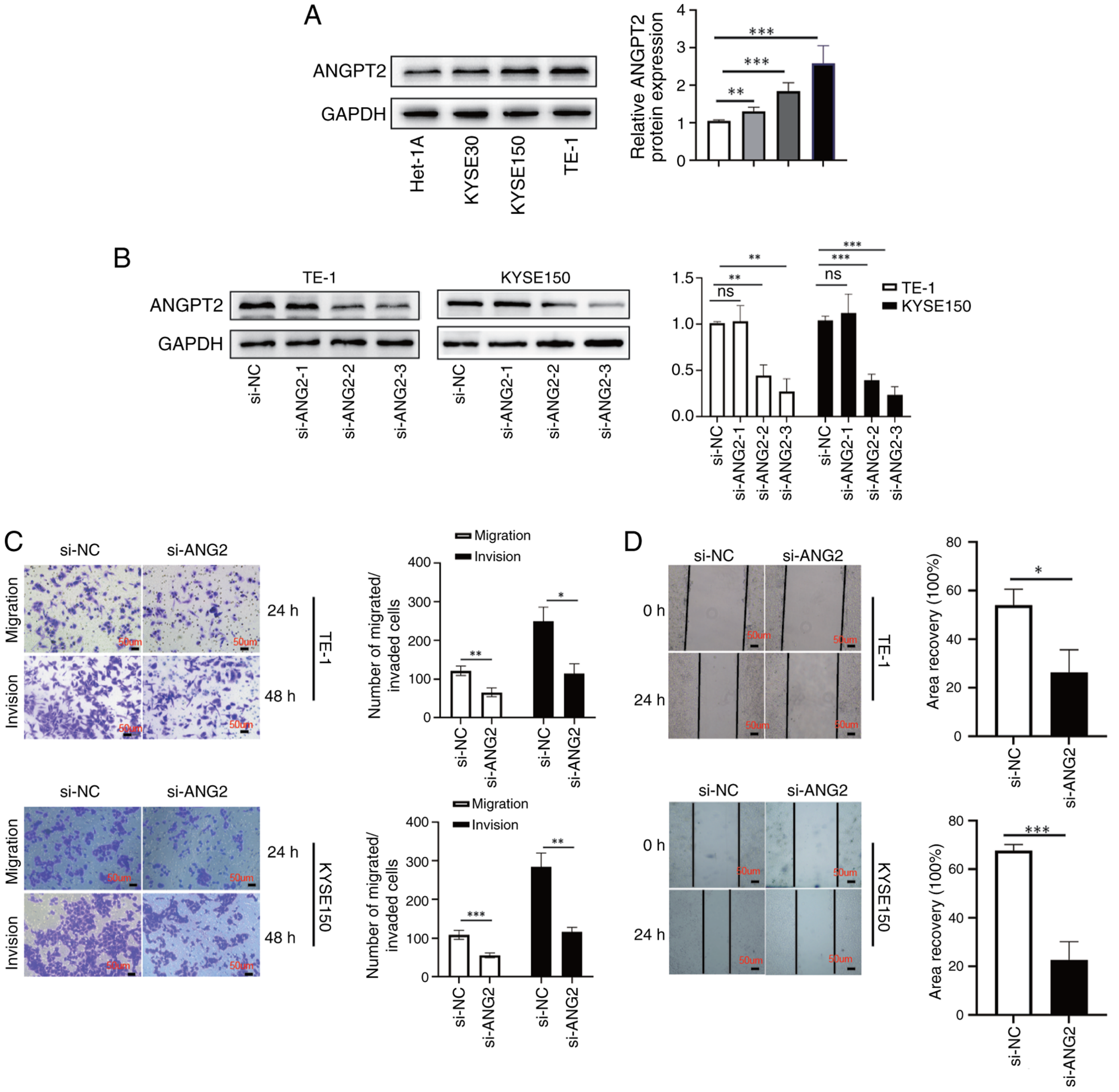

Figure S2. Expression characteristics of ANGPT2 in distinct cell subsets of the esophageal cancer TME. This plot depicts the ANGPT2 expression profile in 8 distinct TME cell populations analyzed from 208,659 single-cell transcriptomes of 60 patients with ESCC in the GSE160269 dataset, with the samples derived from primary ESCC tissues of patients who did not receive neoadjuvant therapy; the X-axis represents the number of single cells, the Y-axis represents the ANGPT2 gene expression level, and different colors correspond to different TME cell types, specifically red for T cells (adaptive immune cells mediating cellular immunity), purple for FRC (stromal cells regulating T cell localization), blue for pericytes (vascular support cells involved in angiogenesis), dark blue for epithelial cells (normal or cancerous esophageal epithelial cells), yellow for fibroblasts (stromal cells promoting extracellular matrix remodeling), gray for endothelial cells (vascular endothelial cells, the dominant TME population), green for B cells (adaptive immune cells involved in humoral immunity) and pink for myeloid cells (innate immune cells such as macrophages and dendritic cells). ANGPT2, angiopoietin-2; TME, tumor microenvironment; ESCC, esophageal squamous cell carcinoma; FRC, fibroblastic reticular cells.

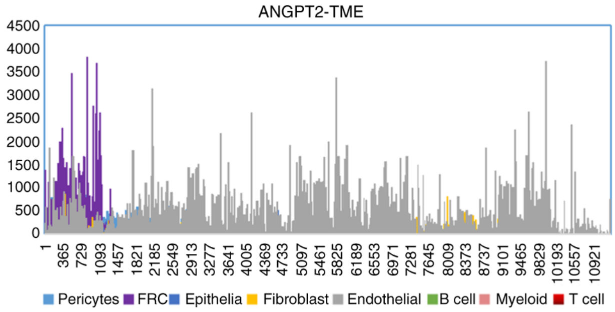

Figure S3. Dual-luciferase reporter gene assay to verify the regulation of long non-coding RNA MAPKAPK5-AS1/SNHG1 on hsa-miR-145-5p and the regulation of hsa-miR-145-5p on ANGPT2. (A-C) Sanger sequencing electropherograms verifying the sequence accuracy of WT and MUT constructs. The targeted mutation of the binding region (AACTGGA to TTGACCT) is marked with red circles in the MUT sequences: (A) ANGPT2 3'UTR (WT/MUT), (B) MAPKAPK5-AS1 (WT/MUT), (C) SNHG1 (WT/MUT). (D) hsa-miR-145-5p directly binds to the 3'UTR of ANGPT2 and negatively regulates ANGPT2 expression in dual-luciferase reporter assays. (E and F) Long non-coding RNAs MAPKAPK5-AS1 (E) and SNHG1 (F) directly bind to hsa-miR-145-5p and inhibit its activity in dual-luciferase reporter assays. All dual-luciferase assays were performed in triplicate, and data are presented as the mean ± SD. *P<0.05 and **P<0.01. ns, not significant; miR, microRNA; ANGPT2, angiopoietin-2; WT, wild-type; MUT, mutant; UTR, untranslated region.

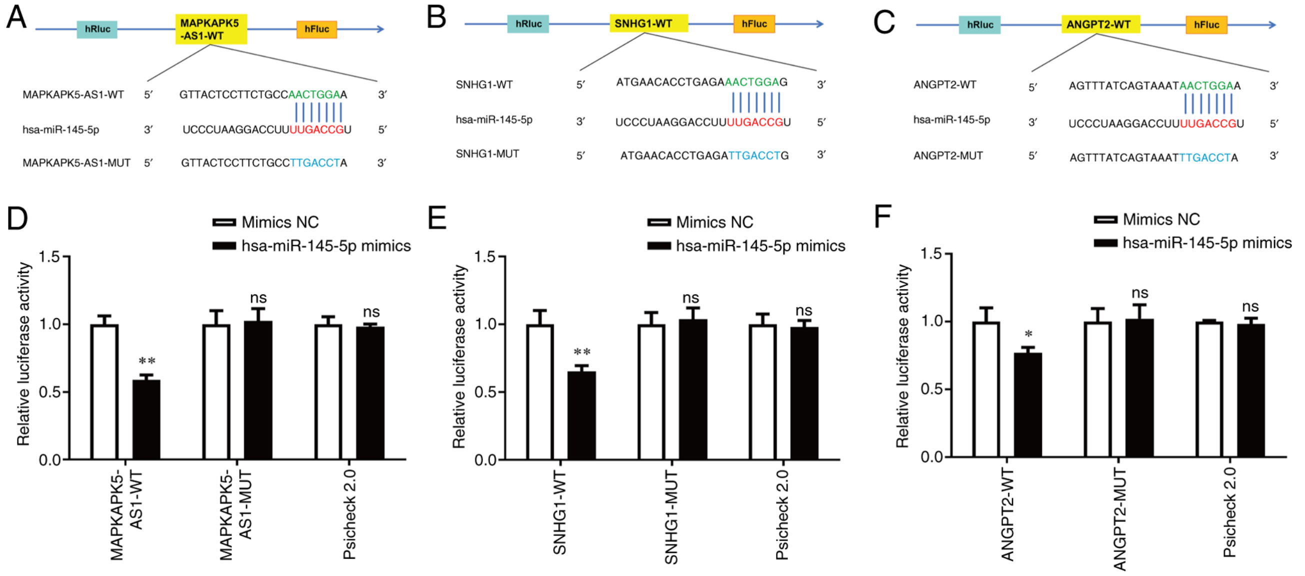

Supplement: Supporting Data [file Supplementary_Data1.pdf]
