# Supplementary material for: Unveiling the role of ANGPT2 in esophageal cancer: A prognostic factor and potential oncogene
Source: Oncol Rep. 2026 Mar 12;55(5):93. doi: 10.3892/or.2026.9098 (PMC13007277; doi:10.3892/or.2026.9098)
Supplement: Supporting Data [file Supplementary_Data2.pdf]

Table SI. Correlation analysis between angiopoietin-2 and immune checkpoints in esophageal cancer determined by The Cancer Genome Atlas database.

Gene	cor	P-value
TGFBR2	0.447878164	3.39E-09
CD200	0.391281767	3.31E-07
BTN2A1	0.375189998	1.05E-06
PVR	0.37275972	1.25E-06
TNFRSF4	0.331972829	1.78E-05
NT5E	0.324015857	2.87E-05
CD28	0.303681687	9.21E-05
HLA-DMB	0.294920268	0.000148558
CD80	0.285244319	0.000247566
CD4	0.281380149	0.000302099
MICB	0.271131208	0.000505458
CASP10	0.255522107	0.001067281
TNFRSF14	0.249391372	0.001414576
CASP8	0.246667542	0.001599801
ADORA2A	0.241615048	0.002003125
CD40LG	0.239148751	0.00217746
HRH4	0.236115423	0.002487352
HLA-DMA	0.235295197	0.002637228
HAVCR2	0.234880271	0.002684635
CCL2	0.233034277	0.002905058
TNFSF4	0.231414091	0.003111891
TNFRSF9	0.22530876	0.004016474
ICOS	0.219609885	0.005067895
TNFRSF10B	0.218723582	0.005251951
CASP6	0.216344112	0.005776092
CASP7	0.210825885	0.007175885
PDCD1LG2	0.207943988	0.008020965
KLRG1	0.199902337	0.010864974
HMOX1	0.181744409	0.020749468
TNFSF14	0.177454023	0.023992941
CASP3	0.168209935	0.032490393
CTLA4	0.166832496	0.033953445
TNFSF9	-0.163273169	0.037995107
CD47	0.158302531	0.044313217
CD48	0.151195169	0.054857387
PTPN11	0.150526207	0.055948782
CD247	0.148253996	0.059790688
CD276	0.146859621	0.062254086
ALK	-0.146592987	0.062681369
CD86	0.146182192	0.063480627
FOXP3	0.140844613	0.073853512
TNFRSF18	-0.140655498	0.074244881
LGALS9	0.1397325	0.076179212
FADD	-0.137412305	0.081222275
FLOT1	0.136912701	0.08234264
CD96	0.130869466	0.096903641
PDCD1	0.125958104	0.110184044
CD24	0.11096998	0.159625538
TIGIT	0.1083647	0.169701145
PSMB8	0.107001373	0.175158748
KIR3DL1	0.102297917	0.195196575
BTLA	0.100977897	0.200832833
CD244	0.097743172	0.215696521
IFNG	0.094161788	0.233318724
PTPN6	-0.093150766	0.238125307
TUBB	-0.092586242	0.240991266
TRIM39	0.089343049	0.257923308

PSMB9	0.089275306	0.258285501
CD160	0.087209146	0.26950082
TNFRSF10A	0.080573162	0.307741337
VTCN1	-0.076864236	0.330596595
IDO1	0.067360467	0.393995223
CD274	0.051071115	0.518263357
DAXX	-0.046916215	0.552900073
OR2H2	-0.036730096	0.642623154
ICOSLG	0.02815989	0.721781731
CD70	0.024820728	0.753640562
CD40	0.02462879	0.755485154
CD27	0.01302499	0.869203331
LAG3	-0.008531376	0.914113774
